# On eccentricity-based entropy measures for dendrimers

**DOI:** 10.1016/j.heliyon.2021.e07762

**Published:** 2021-08-16

**Authors:** Rongbing Huang, Muhammad Kamran Siddiqui, Shazia Manzoor, Sarfraz Ahmad, Murat Cancan

**Affiliations:** a School of Computer Science Chengdu University, Chengdu, China; b Department of Mathematics, COMSATS University Islamabad, Lahore Campus, Pakistan; c Faculty of Education, Van Yuzuncu Yil University, Van, Turkey

**Keywords:** Entropy, Eccentricity-based indices, Molecular graph, Cyclotriphosphazene-based dendrimer

## Abstract

The eccentricity-based entropy inspired by Shannon's entropy approach is the information-theoretic quantity to figure out the structural information of complex networks. The investigation for advance biomedical utilization of dendrimers has improved the synthesis of radical based molecules. Categorically, attaining radical dendrimers has initiated their use in different fields such as anti-tumor agents and as magnetic resonance imaging. The use of radical dendrimers has increased the possibility of establishing new kinds of devices based on para-magnetic axioms of organic radicals. In this article, we discussed dendrimer based on cyclotriphosphazene ($N_{3}P_{3}$) which has balanced edge groups and these are examined by EPR temperature spectrum. Firstly, we computed eccentricity-based indices and then we computed eccentricity based entropies by developing an acquaintance between these indices and their entropies. Moreover we presented our computed result numerically and graphically which leads to good importance of our contribution.

## 1 Introduction

Dendrimers belong to the new class of polymeric materials identified by aggregation of covenant molecular association and immense figure of functional groups, which can form them potential aspirant not only in the field of medicine but also in the field of engineering. Dendrimers are abide by three distinct domains. The size of dendrimers (branched wedges) depends upon the number of monomer layers assembled and every layer is known as a generation [1]. A lot of experiments have performed on these polymers and these experiments proved that the unambiguous dimensional structures and topological constructions of these polymers have many applications in the field of medicine [2]. Recently, dendrimers have accomplished great attention of scientists due to their exceptional chemical and physical properties. In [3], broad scope of dendrimers have been discussed in the different fields of sciences like biology, chemistry, physics, engineering, and medicine [4].

A topological descriptor is also known as graph theoretic index and it is a numerical quantity connected with molecular graph structure and it corresponds different chemical reactivity and physical properties with chemical structures. It is to b noted that topological descriptors of two homomorphic graphs are the same. Among the huge spectrum of topological descriptors [5], [6], [7], [8], [9], [10], [11], [12], [13], distance based topological descriptors have achieved great attention due to their enormous utilization in structure-activity relationship, structure property relationship, and in isomer discrimination.

In this article, we will consider a simple connected molecular graph D in which V(D) and E(D) will represent the vertex set and the edge set respectively. The vertices and edges of D are correlated with atoms and chemical bonds between atoms respectively. Any two vertices of the graph D are the neighboring vertices if and only if they share a common edge. The set of neighboring vertices of *a* is described as $N_{a}=\{z\in V(D):az\in E(D)\}$. The degree of *a* is specified as the cardinality of the set of neighboring vertices of *a* and is denoted by $\hbar_{a}$. The addition of the degrees of adjoining vertices of *a* is $A_{a}=\sum_{z\in N_{a}}\hbar_{a}$. A path containing *n* vertices is described as a graph having $\left\{a_{i}:1\leq i\leq n\right\}$ and $\left\{a_{i}a_{i+1}:1\leq i\leq n-1\right\}$ as the vertex set and the edge set. The distance between any two vertices of D is specified as the range of the shortest path between those vertices. The maximum distance between *a* and any other vertex *z* in D is the eccentricity of the vertex *a*. During his research work, Harold Wiener [14] defined a new quantity named as “Wiener index”. He investigated exquisite correlations between Wiener index and different physico-chemical properties of organic compounds.

Uncertainty is ubiquitous. It appears due to have a few information than the total information needed to characterize a system and its surroundings. It is so closely correlated with information that the amount of removed uncertainty is the same as the information provided by an experiment. In 1948, Claude Shannon [15] introduced a measure of uncertainty recognized as entropy. Though Shannon proposed entropy measure for problems in communication theory, later it has been used in graphs and chemical networks [16], [17]. The entropy measure has found extensive applications in various disciplines such as engineering, physical and biological sciences [18], [19], [20]. This remarkable achievement of entropy is because of having the ability to compute uncertainty occur in probabilistic systems. In the literature, many entropies are computed by using characteristic polynomials, degree of vertices, and the order of the graphs [21], [22], [23], [24]. In the last few years, graph entropies are computed which are grounded on independent sets, matchings, and degree of vertices [25]. Dehmer and Mowshowits introduced some relations between complexity of the graphs as well as Hosoya entropy. For further study, see [15], [16], [22], [26], [27], [28], [29], [30], [31], [32], [33], [34], [35].

## 2 Distance-based topological descriptors

• **Eccentric-connectivity index**

In 1947, Sharma et al. [36] introduced the eccentric connectivity index ϱ(D) as the sum of the product of eccentricity and the degree of every vertex in the molecular graph with *n* vertices.

$$\varrho (D)=\sum_{a\in V(D)}\upsilon (a)\hbar_{a}$$

It is the modified version of an adjacency distance based topological index. This index takes into deliberation the existence as well as relative position of hetero-atoms in the underlying molecular graph. It is also used for mathematical modeling of biological activities of disparate nature. For further details, interested readers are referred to see [37], [38], [39], [40], [41], [42] for chemical meaning and for mathematical properties see [43].

• **Total Eccentricity index**

The total eccentricity is defined as the addition of eccentricity of all the vertices.

$$\varsigma (D)=\sum_{a\in V(D)}\upsilon (a)$$

For further details about this index see [44], [45], [46], [47]

• **First Zagreb eccentric index**

Gutman and Trinajstic introduced the Zagreb type indices [48]. Later, Ghorbani proposed the modified version of first Zagreb index in terms of eccentricity [49] as given below:

$$MM_{1}(D)=\sum_{a\in V(D)}{\left(\upsilon (a)\right)}^{2}$$

• **Augmented eccentric-connectivity index**

Gupta et al. [50] suggested the augmented eccentric-connectivity index of a graph D and it is specified as:

$$Aug_{\upsilon }(D)=\sum_{a\in V(D)}\frac{P(a)}{\upsilon (a)}$$

where P is the product of degrees of all the neighboring vertices of *a*. To study different properties of augmented eccentric-connectivity index see [51], [52], [53], [54].

• **Modified eccentric-connectivity index**

The modified eccentric-connectivity index is specified as:

$$M_{\varrho }(D)=\sum_{a\in V(D)}A_{a}\upsilon (a)$$

For further study, see [55], [56], [57], [58], [59].

## 3 Eccentricity-based entropies of graph

In 1948, Shannon Claud proposed the basic concept of entropy [15]. Later, entropy was applied to graphs and chemical networks [16], [24]. Dehmer [23] applied the graph entropy based on information functional represented by *λ* as given below.

$$ENT_{\lambda }(D)=-\sum_{i=1}^{n}\frac{\lambda (a_{i})}{\sum_{j=1}^{n}\lambda (a_{j})}\log(\frac{\lambda (a_{i})}{\sum_{j=1}^{n}\lambda (a_{j})})$$

(1) $$ENT_{\lambda }(D)=\log(\sum_{i=1}^{n}\lambda (a_{i}))-\sum_{i=1}^{n}\frac{\lambda (a_{i})}{\sum_{j=1}^{n}\lambda (a_{j})}\log(\lambda (a_{i}))$$

Here, logarithm is considered to have base *e*.

Now we introduce the definition of eccentricity based entropies by utilizing a new information functional.

• **Eccentric-connectivity entropy**

For any vertex $a_{i}\in V(D)$, let $\lambda (a_{i})=\upsilon (a_{i})\cdot \hbar_{a_{i}}$, then eccentric-connectivity entropy by using equation (1) is:

(2) $$ENT_{\varrho }(D)=\log(\varrho (D))-\frac{1}{\varrho (D)}\sum_{i=1}^{n}(\upsilon (a_{i})\cdot \hbar_{a_{i}})\log(\upsilon (a_{i})\cdot \hbar_{a_{i}})$$

• **Total eccentric-connectivity entropy**

For any vertex $a_{i}\in V(D)$, let $\lambda (a_{i})=\upsilon (a_{i})$, then total eccentricity entropy by using equation (1) is:

(3) $$ENT_{\varsigma }(D)=\log(\varsigma (D))-\frac{1}{\varsigma (D)}\sum_{i=1}^{n}\upsilon (a_{i})\log\upsilon (a_{i})$$

• **First Zagreb eccentric entropy**

For any vertex $a_{i}\in V(D)$, let $\lambda (a_{i})=\upsilon {\left(a_{i}\right)}^{2}$, then first Zagreb eccentric entropy by using equation (1) is:

(4) $$ENT_{MM_{1}}(D)=\log(MM_{1}(D))-\frac{1}{MM_{1}(D)}\sum_{i=1}^{n}{\left(\upsilon (a_{i})\right)}^{2}\log{\left(\upsilon (a_{i})\right)}^{2}$$

• **Augmented eccentric-connectivity entropy**

For any vertex $a_{i}\in V(D)$, let $\lambda (a_{i})=\frac{P(a_{i})}{\upsilon (a_{i})}$, then augmented eccentric-connectivity entropy by using equation (1) is:

(5) $$ENT_{Aug_{\upsilon }}(D)=\log(Aug_{\upsilon }(D))-\frac{1}{Aug_{\upsilon }(D)}\sum_{i=1}^{n}\frac{P(a_{i})}{\upsilon (a_{i})}\log\frac{P(a_{i})}{\upsilon (a_{i})}$$

• **Modified eccentric-connectivity entropy**

For any vertex $a_{i}\in V(D)$, let $\lambda (a_{i})=A_{a_{i}}\cdot \upsilon (a_{i})$, then modified eccentric-connectivity entropy by using equation (1) is:

(6) $$ENT_{M_{\varrho }}(D)=\log(M_{\varrho }(D))-\frac{1}{M_{\varrho }(D)}\sum_{i=1}^{n}(A_{a_{i}}\cdot \upsilon (a_{i}))\log(A_{a_{i}}\cdot \upsilon (a_{i}))$$

## 4 Structure of phosphorus containing dendrimer Cyclotriphosphazene $N_{3}P_{3}$

**Figure 1:**
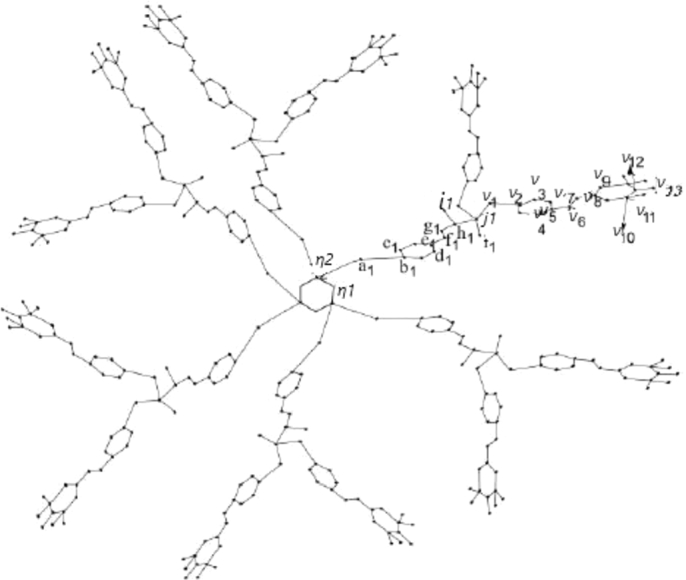
An illustration of molecular graph G(x) for the first generation.

**Figure 2:**
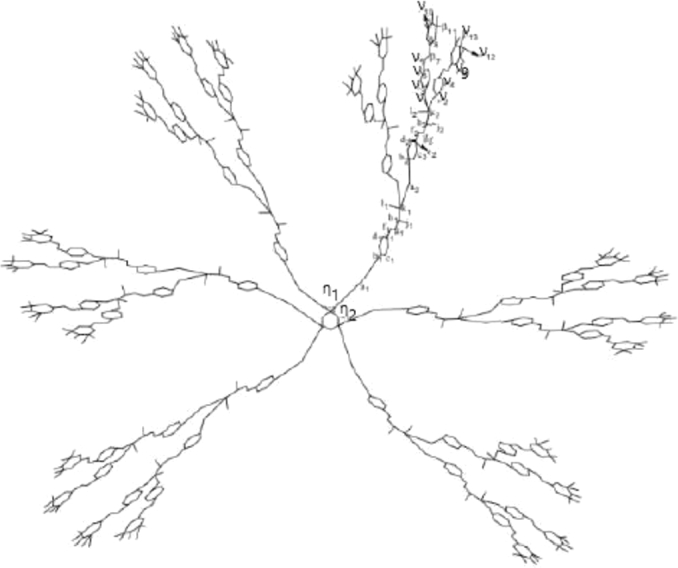
An illustration of molecular graph G(x) for the second generation.

Let G(x) represent the molecular graph of phosphorus having dendrimer Cyclotriphosphazene $N_{3}P_{3}$ in which *x* represents the generation stage of G(x). Fig. 1 and Fig. 2 respectively show the first and the second generation stage of G(x) [60]. It is easy to see that $|V(G(x))|=216\times 2^{x}-78$ and $|E(G(x))|=99\times 2^{x}-72$. To compute eccentricity based descriptors and respective entropies of G(x), we will use computational arguments. For this, we will divide the vertex set of G(x) into three representatives X, Y, and Z, where $X=\{\eta_{k},1\leq k\leq 2\}$, $Y=\{\nu_{k},1\leq k\leq 13\}$, and $Z=\{a_{k},b_{k},c_{k},d_{k},e_{k},f_{k},g_{k},h_{k},i_{k},j_{j},l_{k}:1\leq k\leq n\}$, these three groups of representatives can be seen in Fig. 1 and Fig. 2.

Table 1 and Table 2 represent the degree, $P_{a}$, $A_{a}$, and υ(a) for each a∈X,Y and for each a∈Z respectively.

**Table 1:** The degree, $P_{a}$, $A_{a}$, and *υ*(*a*) and frequencies for each a∈X,Y.

Representative	*ℏ*_*a*_	$A_{a}$	$P_{a}$	*υ*_*a*_	Frequencies
*η*_1_	2	8	16	15 + 9*x*	3
*η*_2_	4	8	16	14 + 9*x*	3
*ν*_1_	2	7	12	15 + 9*x*	6 × 2^*x*^
*ν*_2_	3	6	8	16 + 9*x*	6 × 2^*x*^
*ν*_3_	2	5	6	17 + 9*x*	12 × 2^*x*^
*ν*_4_	2	5	6	18 + 9*x*	12 × 2^*x*^
*ν*_5_	3	6	8	19 + 9*x*	6 × 2^*x*^
*ν*_6_	2	5	6	20 + 9*x*	6 × 2^*x*^
*ν*_7_	2	5	6	21 + 9*x*	6 × 2^*x*^
*ν*_8_	3	6	8	22 + 9*x*	6 × 2^*x*^
*ν*_9_	2	7	12	23 + 9*x*	12 × 2^*x*^
*ν*_10_	4	7	6	24 + 9*x*	12 × 2^*x*^
*ν*_11_	1	4	4	25 + 9*x*	24 × 2^*x*^
*ν*_12_	3	9	16	25 + 9*x*	6 × 2^*x*^
*ν*_13_	1	3	3	26 + 9*x*	6 × 2^*x*^

**Table 2:** The degree, $P_{a}$, $A_{a}$, and *υ*(*a*) and frequencies for each a∈Z.

Representative	*ℏ*_*a*_	$A_{a}$	$P_{a}$	*υ*_*a*_	Frequencies
*a*_*k*_	2	7	12	6 + 9(*x* + *k*)	2^*k*^ × 3
*b*_*k*_	3	6	8	7 + 9(*x* + *k*)	2^*k*^ × 3
*c*_*k*_	2	5	6	8 + 9(*x* + *k*)	2^*k*+1^ × 3
*d*_*k*_	2	5	6	9 + 9(*x* + *k*)	2^*k*+1^ × 3
*e*_*k*_	3	6	8	10 + 9(*x* + *k*)	2^*k*^ × 3
*f*_*k*_	2	5	6	11 + 9(*x* + *k*)	2^*k*^ × 3
*g*_*k*_	2	5	6	12 + 9(*x* + *k*)	2^*k*^ × 3
*h*_*k*_	3	7	8	13 + 9(*x* + *k*)	2^*k*^ × 3
*i*_*k*_	1	3	3	14 + 9(*x* + *k*)	2^*k*^ × 3
*j*_*k*_	4	8	12	14 + 9(*x* + *k*)	2^*k*^ × 3
*l*_*k*_	1	4	4	15 + 9(*x* + *k*)	2^*k*^ × 3

### 4.1 Entropy measure for the molecular graph G(x)

In this portion, we will estimate the entropies of G(x).

• **Eccentric-connectivity entropy of**G(x)

By using Table 1 and Table 2, we computed the eccentric-connectivity descriptor as:

$$\varrho (G(x))=5400x\times 2^{x}+6150\times 2^{x}-1296x+6$$

By substituting the value of above calculated index in equation (2), we get the eccentric-connectivity entropy as:

$$ENT_{\varrho }(G(x))=\log(5400x\times 2^{x}+6150\times 2^{x}-1296x+6)-\frac{1}{5400x\times 2^{x}+6150\times 2^{x}-1296x+6}(6(15+9x)\log(30+18x)+12(14+9x)\log(56+36x)+(2^{x}\times 12)(15+9x)\log(30+18x)+(2^{x}\times 18)(16+9x)\log(48+27x)+(2^{x}\times 24)(17+9x)\log(34+18x)+(2^{x}\times 24)(18+9x)\log(36+18x)+(2^{x}\times 18)(19+9x)\log(57+27x)+(2^{x}\times 12)(20+9x)\log(40+18x)+(2^{x}\times 12)(21+9x)\log(42+18x)+(2^{x}\times 18)(22+9x)\log(66+27x)+(2^{x}\times 24)(23+9x)\log(46+18x)+(2^{x}\times 48)(24+9x)\log(96+36x)+(2^{x}\times 24)(25+9x)\log(25+9x)+(2^{x}\times 36)(25+9x)\log(75+27x)+(2^{x}\times 6)(26+9x)\log(26+9x)+\sum_{k=1}^{x}(6\cdot 2^{k}(6+9k+9x))\log(\sum_{k=1}^{x}(2(6+9k+9x)))+\sum_{k=1}^{x}(9\cdot 2^{k}(7+9k+9x))\log(\sum_{k=1}^{x}(3(7+9k+9x)))+\sum_{k=1}^{x}(12\cdot 2^{k}(8+9k+9x))\log(\sum_{k=1}^{x}(2(8+9k+9x)))+\sum_{k=1}^{x}(12\cdot 2^{k}(9+9k+9x))\log(\sum_{k=1}^{x}(2(9+9k+9x)))+\sum_{k=1}^{x}(9\cdot 2^{k}(10+9k+9x))\log(\sum_{k=1}^{x}(3(10+9k+9x)))+\sum_{k=1}^{x}(6\cdot 2^{k}(11+9k+9x))\log(\sum_{k=1}^{x}(2(11+9k+9x)))+\sum_{k=1}^{x}(6\cdot 2^{k}(12+9k+9x))\log(\sum_{k=1}^{x}(2(12+9k+9x)))+\sum_{k=1}^{x}(9\cdot 2^{k}(13+9k+9x))\log(\sum_{k=1}^{x}(3(13+9k+9x)))+\sum_{k=1}^{x}(3\cdot 2^{k}(14+9k+9x))\log(\sum_{k=1}^{x}(14+9k+9x))+\sum_{k=1}^{x}(12\cdot 2^{k}(14+9k+9x))\log(\sum_{k=1}^{x}(4(14+9k+9x)))+\sum_{k=1}^{x}(3\cdot 2^{k}(15+9k+9x))\log(\sum_{k=1}^{x}(15+9k+9x)))$$

• **Total eccentric-connectivity entropy of**G(x)

By using Table 1 and Table 2, we computed the total eccentric-connectivity descriptor as:

$$\varrho (G(x))=2430x\times 2^{x}+2838\times 2^{x}-594x-33$$

By substituting the value of above calculated index in equation (3), we get the total eccentric-connectivity entropy as:

$$ENT_{\varsigma }(G(x))=\log(2430x\times 2^{x}+2838\times 2^{x}-594x-33)-\frac{1}{2430x\times 2^{x}+2838\times 2^{x}-594x-33}(3(15+9x)\log(15+9x)+3(14+9x)\log(14+9x)+(2^{x}\times 6)(15+9x)\log(15+9x)+(2^{x}\times 6)(16+9x)\log(16+9x)+(2^{x}\times 12)(17+9x)\log(17+9x)+(2^{x}\times 12)(18+9x)\log(18+9x)+(2^{x}\times 6)(19+9x)\log(19+9x)+(2^{x}\times 6)(20+9x)\log(20+9x)+(2^{x}\times 6)(21+9x)\log(21+9x)+(2^{x}\times 6)(22+9x)\log(22+9x)+(2^{x}\times 12)(23+9x)\log(23+9x)+(2^{x}\times 12)(24+9x)\log(24+9x)+(2^{x}\times 24)(25+9x)\log(25+9x)+(2^{x}\times 12)(25+9x)\log(25+9x)+(2^{x}\times 6)(26+9x)\log(26+9x)+\sum_{k=1}^{x}(3\cdot 2^{k}(6+9k+9x))\log(\sum_{k=1}^{x}(6+9k+9x))+\sum_{k=1}^{x}(3\cdot 2^{k}(7+9k+9x))\log(\sum_{k=1}^{x}(7+9k+9x))+\sum_{k=1}^{x}(6\cdot 2^{k}(8+9k+9x))\log(\sum_{k=1}^{x}(8+9k+9x))+\sum_{k=1}^{x}(6\cdot 2^{k}(9+9k+9x))\log(\sum_{k=1}^{x}(9+9k+9x))+\sum_{k=1}^{x}(3\cdot 2^{k}(10+9k+9x))\log(\sum_{k=1}^{x}(10+9k+9x))+\sum_{k=1}^{x}(3\cdot 2^{k}(11+9k+9x))\log(\sum_{k=1}^{x}(11+9k+9x))+\sum_{k=1}^{x}(3\cdot 2^{k}(12+9k+9x))\log(\sum_{k=1}^{x}(12+9k+9x))+\sum_{k=1}^{x}(3\cdot 2^{k}(13+9k+9x))\log(\sum_{k=1}^{x}(13+9k+9x))+2\sum_{k=1}^{x}(3\cdot 2^{k}(14+9k+9x))\log(\sum_{k=1}^{x}(14+9k+9x))+\sum_{k=1}^{x}(3\cdot 2^{k}(15+9k+9x))\log(\sum_{k=1}^{x}(15+9k+9x)))$$

• **First Zagreb eccentric entropy of**G(x)

By using Table 1 and Table 2, we computed the first Zagreb eccentric descriptor as:

$$MM_{1}(G(x))=33534x^{2}\cdot 2^{x}+53244x\cdot 2^{x}-5346x^{2}+72618\cdot 2^{x}-594x-11181$$

By substituting the value of the above calculated index in equation (4), we get the first Zagreb eccentric entropy as:

$$ENT_{MM_{1}}(G(x))=\log(33534x^{2}\cdot 2^{x}+53244x\cdot 2^{x}-5346x^{2}+72618\cdot 2^{x}-594x-11181)-\frac{1}{33534x^{2}\cdot 2^{x}+53244x\cdot 2^{x}-5346x^{2}+72618\cdot 2^{x}-594x-11181}(3{\left(15+9x\right)}^{2}\log{\left(15+9x\right)}^{2}+3{\left(14+9x\right)}^{2}\log{\left(14+9x\right)}^{2}+(2^{x}\times 6){\left(15+9x\right)}^{2}\log{\left(15+9x\right)}^{2}+(2^{x}\times 6){\left(16+9x\right)}^{2}\log{\left(16+9x\right)}^{2}+(2^{x}\times 12){\left(17+9x\right)}^{2}\log{\left(17+9x\right)}^{2}+(2^{x}\times 12){\left(18+9x\right)}^{2}\log{\left(18+9x\right)}^{2}+(2^{x}\times 6){\left(19+9x\right)}^{2}\log{\left(19+9x\right)}^{2}+(2^{x}\times 6){\left(20+9x\right)}^{2}\log{\left(20+9x\right)}^{2}+(2^{x}\times 6){\left(21+9x\right)}^{2}\log{\left(21+9x\right)}^{2}+(2^{x}\times 6){\left(22+9x\right)}^{2}\log{\left(22+9x\right)}^{2}+(2^{x}\times 12){\left(23+9x\right)}^{2}\log{\left(23+9x\right)}^{2}+(2^{x}\times 12){\left(24+9x\right)}^{2}\log{\left(24+9x\right)}^{2}+(2^{x}\times 24){\left(25+9x\right)}^{2}\log{\left(25+9x\right)}^{2}+(2^{x}\times 12){\left(25+9x\right)}^{2}\log{\left(25+9x\right)}^{2}+(2^{x}\times 6){\left(26+9x\right)}^{2}\log{\left(26+9x\right)}^{2}+\sum_{k=1}^{x}(3\cdot 2^{k}{\left(6+9k+9x\right)}^{2})\log(\sum_{k=1}^{x}{\left(6+9k+9x\right)}^{2})+\sum_{k=1}^{x}(3\cdot 2^{k}{\left(7+9k+9x\right)}^{2})\log(\sum_{k=1}^{x}{\left(7+9k+9x\right)}^{2})+\sum_{k=1}^{x}(6\cdot 2^{k}{\left(8+9k+9x\right)}^{2})\log(\sum_{k=1}^{x}{\left(8+9k+9x\right)}^{2})+\sum_{k=1}^{x}(6\cdot 2^{k}{\left(9+9k+9x\right)}^{2})\log(\sum_{k=1}^{x}{\left(9+9k+9x\right)}^{2})+\sum_{k=1}^{x}(3\cdot 2^{k}{\left(10+9k+9x\right)}^{2})\log(\sum_{k=1}^{x}{\left(10+9k+9x\right)}^{2})+\sum_{k=1}^{x}(3\cdot 2^{k}{\left(11+9k+9x\right)}^{2})\log(\sum_{k=1}^{x}{\left(11+9k+9x\right)}^{2})$$

$$ENT_{MM_{1}}(G(x))=+\sum_{k=1}^{x}(3\cdot 2^{k}{\left(12+9k+9x\right)}^{2})\log(\sum_{k=1}^{x}{\left(12+9k+9x\right)}^{2})+\sum_{k=1}^{x}(3\cdot 2^{k}{\left(13+9k+9x\right)}^{2})\log(\sum_{k=1}^{x}{\left(13+9k+9x\right)}^{2})+2\sum_{k=1}^{x}(3\cdot 2^{k}{\left(14+9k+9x\right)}^{2})\log(\sum_{k=1}^{x}{\left(14+9k+9x\right)}^{2})+\sum_{k=1}^{x}(3\cdot 2^{k}{\left(15+9k+9x\right)}^{2})\log(\sum_{k=1}^{x}{\left(15+9k+9x\right)}^{2}))$$

• **Augmented eccentric-connectivity entropy of**G(x)

By using Table 1 and Table 2, we computed the augmented eccentric-connectivity descriptor as:

$$Aug_{\upsilon }(G(x))=\frac{48}{15+9x}+\frac{48}{14+9x}+\frac{2^{x}\cdot 72}{15+9x}+\frac{2^{x}\cdot 48}{16+9x}+\frac{2^{x}\cdot 72}{17+9x}+\frac{2^{x}\cdot 72}{18+9x}+\frac{2^{x}\cdot 48}{19+9x}+\frac{2^{x}\cdot 36}{20+9x}+\frac{2^{x}\cdot 36}{21+9x}+\frac{2^{x}\cdot 144}{23+9x}+\frac{2^{x}\cdot 72}{24+9x}+\frac{2^{x}\cdot 96}{25+9x}+\frac{2^{x}\cdot 96}{25+9x}+\frac{2^{x}\cdot 18}{26+9x}+\sum_{k=1}^{x}(\frac{36\cdot 2^{k}}{6+9k+9x})+\sum_{k=1}^{x}(\frac{24\cdot 2^{k}}{7+9k+9x})+\sum_{k=1}^{x}(\frac{36\cdot 2^{k}}{8+9k+9x})+\sum_{k=1}^{x}(\frac{36\cdot 2^{k}}{9+9k+9x})+\sum_{k=1}^{x}(\frac{24\cdot 2^{k}}{10+9k+9x})+\sum_{k=1}^{x}(\frac{18\cdot 2^{k}}{11+9k+9x})+\sum_{k=1}^{x}(\frac{18\cdot 2^{k}}{12+9k+9x})+\sum_{k=1}^{x}(\frac{24\cdot 2^{k}}{13+9k+9x})+\sum_{k=1}^{x}(\frac{9\cdot 2^{k}}{14+9k+9x})+\sum_{k=1}^{x}(\frac{36\cdot 2^{k}}{14+9k+9x})+\sum_{k=1}^{x}(\frac{12\cdot 2^{k}}{15+9k+9x})$$

By substituting the value of above calculated index in equation (5), we get the augmented eccentric-connectivity entropy as:

$$ENT_{Aug_{\upsilon }}(G(x))=\log(Aug_{\upsilon }(G(x)))-\frac{1}{Aug_{\upsilon }(G(x))}(\frac{48}{15+9x}\log(\frac{16}{15+9x})+\frac{48}{14+9x}\log(\frac{16}{14+9x})+\frac{2^{x}\cdot 72}{15+9x}\log(\frac{12}{15+9x})+\frac{2^{x}\cdot 48}{16+9x}\log(\frac{8}{16+9x})+\frac{2^{x}\cdot 72}{17+9x}\log(\frac{6}{17+9x})+\frac{2^{x}\cdot 72}{18+9x}\log(\frac{6}{18+9x})+\frac{2^{x}\cdot 48}{19+9x}\log(\frac{8}{19+9x})+\frac{2^{x}\cdot 36}{20+9x}\log(\frac{6}{20+9x})+\frac{2^{x}\cdot 36}{21+9x}\log(\frac{6}{21+9x})+\frac{2^{x}\cdot 144}{23+9x}\log(\frac{12}{23+9x})+\frac{2^{x}\cdot 72}{24+9x}\log(\frac{6}{24+9x})+\frac{2^{x}\cdot 96}{25+9x}\log(\frac{4}{25+9x})+\frac{2^{x}\cdot 96}{25+9x}\log(\frac{16}{25+9x})+\frac{2^{x}\cdot 18}{26+9x}\log(\frac{3}{26+9x})+\sum_{k=1}^{x}(\frac{36\cdot 2^{k}}{6+9k+9x})\log(\sum_{k=1}^{x}(\frac{12}{6+9k+9x}))+\sum_{k=1}^{x}(\frac{24\cdot 2^{k}}{7+9k+9x})\log(\sum_{k=1}^{x}(\frac{8}{7+9k+9x}))+\sum_{k=1}^{x}(\frac{36\cdot 2^{k}}{8+9k+9x})\log(\sum_{k=1}^{x}(\frac{6}{8+9k+9x}))+\sum_{k=1}^{x}(\frac{36\cdot 2^{k}}{9+9k+9x})\log(\sum_{k=1}^{x}(\frac{6}{9+9k+9x}))+\sum_{k=1}^{x}(\frac{24\cdot 2^{k}}{10+9k+9x})\log(\sum_{k=1}^{x}(\frac{8}{10+9k+9x}))+\sum_{k=1}^{x}(\frac{18\cdot 2^{k}}{11+9k+9x})\log(\sum_{k=1}^{x}(\frac{6}{11+9k+9x}))+\sum_{k=1}^{x}(\frac{18\cdot 2^{k}}{12+9k+9x})\log(\sum_{k=1}^{x}(\frac{6}{12+9k+9x}))+\sum_{k=1}^{x}(\frac{24\cdot 2^{k}}{13+9k+9x})\log(\sum_{k=1}^{x}(\frac{8}{13+9k+9x}))+\sum_{k=1}^{x}(\frac{9\cdot 2^{k}}{14+9k+9x})\log(\sum_{k=1}^{x}(\frac{3}{14+9k+9x}))+\sum_{k=1}^{x}(\frac{36\cdot 2^{k}}{14+9k+9x})\log(\sum_{k=1}^{x}(\frac{12}{14+9k+9x}))+\sum_{k=1}^{x}(\frac{12\cdot 2^{k}}{15+9k+9x})\log(\sum_{k=1}^{x}(\frac{4}{15+9k+9x})))$$

• **Modified eccentric-connectivity entropy of**G(x)

By using Table 1 and Table 2, we computed the modified eccentric-connectivity descriptor as:

$$\varrho (G(x))=14184x\cdot 2^{x}+16108\cdot 2^{x}-3420x+116$$

By substituting the value of above calculated index in equation (6), we get the modified eccentric-connectivity entropy as:

$$ENT_{\varrho }(G(x))=\log(14184x\cdot 2^{x}+16108\cdot 2^{x}-3420x+116)-\frac{1}{14184x\cdot 2^{x}+16108\cdot 2^{x}-3420x+116}(24(15+9x)\log(120+72x)+24(14+9x)\log(112+72x)+(2^{x}\times 42)(15+9x)\log(135+63x)+(2^{x}\times 36)(16+9x)\log(96+54x)+(2^{x}\times 60)(17+9x)\log(85+45x)+(2^{x}\times 60)(18+9x)\log(90+45x)+(2^{x}\times 36)(19+9x)\log(114+54x)+(2^{x}\times 30)(20+9x)\log(100+45x)+(2^{x}\times 30)(21+9x)\log(105+45x)+(2^{x}\times 36)(22+9x)\log(132+54x)+(2^{x}\times 84)(23+9x)\log(161+63x)+(2^{x}\times 84)(24+9x)\log(168+63x)+(2^{x}\times 96)(25+9x)\log(100+36x)+(2^{x}\times 108)(25+9x)\log(225+81x)+(2^{x}\times 18)(26+9x)\log(78+27x)+\sum_{k=1}^{x}(21\cdot 2^{k}(6+9k+9x))\log(\sum_{k=1}^{x}(7(6+9k+9x)))+\sum_{k=1}^{x}(18\cdot 2^{k}(7+9k+9x))\log(\sum_{k=1}^{x}(6(7+9k+9x)))+\sum_{k=1}^{x}(30\cdot 2^{k}(8+9k+9x))\log(\sum_{k=1}^{x}(5(8+9k+9x)))+\sum_{k=1}^{x}(30\cdot 2^{k}(9+9k+9x))\log(\sum_{k=1}^{x}(5(9+9k+9x)))+\sum_{k=1}^{x}(18\cdot 2^{k}(10+9k+9x))\log(\sum_{k=1}^{x}(6(10+9k+9x)))+\sum_{k=1}^{x}(15\cdot 2^{k}(11+9k+9x))\log(\sum_{k=1}^{x}(5(11+9k+9x)))+\sum_{k=1}^{x}(15\cdot 2^{k}(12+9k+9x))\log(\sum_{k=1}^{x}(5(12+9k+9x)))+\sum_{k=1}^{x}(21\cdot 2^{k}(13+9k+9x))\log(\sum_{k=1}^{x}(7(13+9k+9x)))+\sum_{k=1}^{x}(9\cdot 2^{k}(14+9k+9x))\log(\sum_{k=1}^{x}(3(14+9k+9x)))+\sum_{k=1}^{x}(25\cdot 2^{k}(14+9k+9x))\log(\sum_{k=1}^{x}(8(14+9k+9x)))+\sum_{k=1}^{x}(12\cdot 2^{k}(15+9k+9x))\log(\sum_{k=1}^{x}(4(15+9k+9x))))$$

## 5 Comparisons and discussion for G(x)

**Figure 3:**
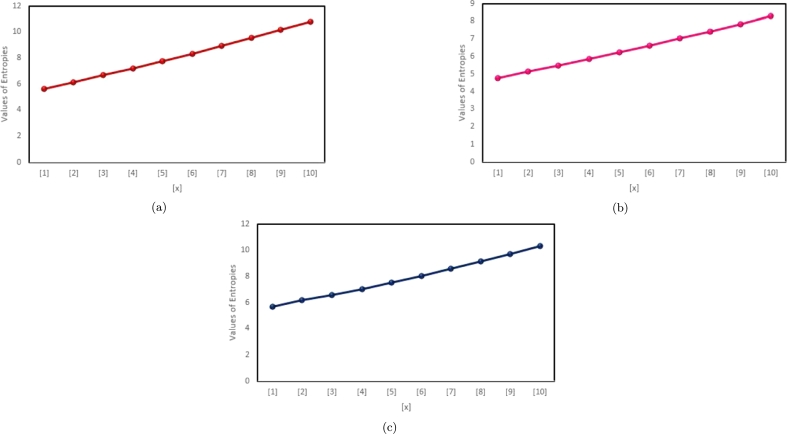
(a) The eccentric-connectivity Entropy, (b) The total eccentric-connectivity entropy, (c) The first Zagreb eccentric entropy, of G(x).

**Figure 4:**
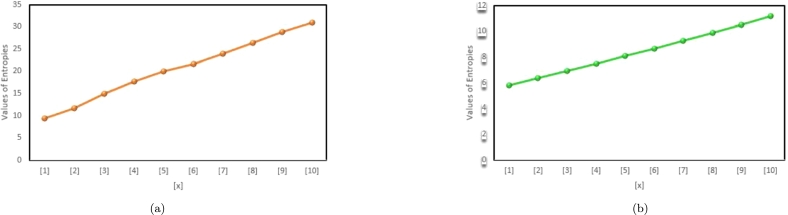
(a) The augmented eccentric connectivity entropy, (b) The modified eccentric connectivity entropy.

The last two decennium have endorsed an excessive growth with respect to applications of information theoretic framework in various branches of science such as biological, physical, engineering and in social sciences. Specifically, this enormous growth has been astonishing in the field of soft computing, molecular biology and information technology. The information theory, led by Claud Shannon, is as significant as when it was proposed. Shannon proposed the concept of entropy to compute upper limits on communication rates in telephonic channels, optical communication and in wireless. The distinguished aspect of entropy is that it empowers the amount of uncertainty in a system. Therefore, our numeric results in Table 3, Table 4 and graphic results in Fig. 3, Fig. 4 might be helpful for the scientists.

**Table 3:** Comparison of $ENT_{\varrho }(G(x))$, $ENT_{\varsigma }(G(x))$, $ENT_{MM_{1}}(G(x))$.

[*x*]	$ENT_{\varrho }(G(x))$	$ENT_{\varsigma }(G(x))$	$ENT_{MM_{1}}(G(x))$
[1]	5.6275	4.7701	5.6868
[2]	6.1513	5.1455	6.1548
[3]	6.6622	5.4911	6.5845
[4]	7.1968	5.8493	7.0425
[5]	7.7556	6.2241	7.5344
[6]	8.3342	6.6137	8.0551
[7]	8.9282	7.0155	8.5987
[8]	9.5344	7.4274	9.1606
[9]	10.1501	7.8475	9.7371
[10]	10.7736	8.2746	10.3255

**Table 4:** Comparison of $ENT_{Aug_{\upsilon }}(G(x))$, and $ENT_{M_{\varrho }}(G(x))$.

[*x*]	$ENT_{Aug_{\upsilon }}(G(x))$	$ENT_{M_{\varrho }}(G(x))$
[1]	9.2876	5.7736
[2]	11.6754	6.3601
[3]	14.9768	6.9157
[4]	17.6817	7.4827
[5]	18.9956	8.0662
[6]	19.9801	8.6644
[7]	20.5844	9.2745
[8]	21.2017	9.8942
[9]	22.4120	10.5217
[10]	23.3694	11.1556

## 6 Conclusion

Among the other topological indices, the eccentricity based indices are of great importance due to their excellent degree of unpredictability of pharmaceutical properties. In this study, we computed eccentric-connectivity index, modified eccentric connectivity index, augmented connectivity index, and first Zagreb eccentric connectivity index, also computed their corresponding entropies for a group of dendrimers which contain phosphorus. We demonstrated our computed results numerically and graphically which could help the scientists to know the physico-chemical properties of this molecular structure. Also we give details comparison of our computed result.

## Declarations

### Author contribution statement

R. Huang, M.K. Siddiqui, S. Manzoor, S. Ahmad, M. Cancan: Conceived and designed the experiments; Performed the experiments; Analyzed and interpreted the data; Contributed reagents, materials, analysis tools or data; Wrote the paper.

### Funding statement

This research did not receive any specific grant from funding agencies in the public, commercial, or not-for-profit sectors.

### Data availability statement

No data was used for the research described in the article.

### Declaration of interests statement

The authors declare no conflict of interest.

### Additional information

No additional information is available for this paper.
